# The Presence of Cough and Tuberculosis: Active Case Finding Outcomes in the Philippines

**DOI:** 10.1155/2019/4578329

**Published:** 2019-08-21

**Authors:** Siwon Lee, Lincoln Lau, Krisha Lim, Jansel Ferma, Warren Dodd, Donald Cole

**Affiliations:** ^1^International Care Ministries Foundation Inc., Philippines; ^2^Dalla Lana School of Public Health, University of Toronto, ON, Canada; ^3^School of Public Health and Health Systems, University of Waterloo, ON, Canada

## Abstract

The results of a tuberculosis (TB) active case finding (ACF) program, implemented by International Care Ministries (ICM) in the Philippines, were examined to understand how the presence of physical symptoms might influence ACF outcomes among extreme low-income Filipinos. ICM health staff implemented symptom screening in villages and suspected cases were referred to the closest rural health unit (RHU) for TB testing. ACF was carried out in Mindanao and the Visayas, across 16 different provinces. All participants were interviewed pre/postprogram, and screening outcomes were collected. A multilevel regression model was constructed to explore the effect of factors on the likelihood of getting tested. A total of 4635 individuals were screened; 1290 (27.8%) were symptom positive and referred. From those referred, 336 (7.2%) were tested for TB and 53 (1.1%) were TB positive. “Cough for more than two weeks” was associated with a 1.09 (95% CI 1.01, 1.15) times increase in likelihood of getting tested. The finding that the presence of cough is associated with higher rate of testing suggests that individuals in these settings might not know or believe that the lack of cough does not equate to lack of TB infection. While technologies and screening algorithms give us the ability to refine the ‘supply' side of the TB screening, addressing the knowledge gap should improve ‘demand'.

## 1. Introduction

Tuberculosis is not a newly emerging disease in the Philippines [1]. Philippines is one of the five countries in the world that share 56% of the global TB patient burden, along with China, India, Indonesia, and Pakistan [2]. Philippines was a pioneer in making state-of-the-art MDR-TB treatment available and the level of awareness of TB in the country is high [3]. Nevertheless, a gap persists in understanding how a TB suspect, experiencing extreme low-income, perceives and responds to common TB symptoms. As a result of their geographic isolation, rural TB patients often face greater financial and transportation burdens associated with accessing healthcare and TB treatment compared to their urban counterparts [4]. More specifically, these financial burdens often include out of pocket expenses for nonmedical costs associated with seeking care, in addition to lost days of work or unemployment from debilitating side effects of the disease and subsequent treatment [5].

According to the WHO, TB symptoms and signs may include cough for two weeks or more, fever, unexplained weight loss, weakness or fatigue, night sweats, and chest pain [6]. Among these symptoms, long-term cough is one of the most commonly identified symptoms by TB patients [7]. Fever and unexplained weight loss are also noted as common symptoms [8]. Based on the Health Behaviour model, an individual's health-seeking behaviour is shaped by an individual's perception of his or her susceptibility to and severity of a specific disease, in addition to barriers to take action [9]. Knowledge about different diseases, accessibility to healthcare facilities, behaviour, and personal background also play a part in an individual's decision making process [8]. According to the model, when an individual is more familiar with a disease, this individual should have greater awareness and capacity to address the disease.

Nevertheless, relying on patients to identify TB via self-reported symptoms is problematic for several reasons. These symptoms are neither specific nor exclusive to TB [10] and their interpretation could vary from person to person, depending on their tolerance [7]. Furthermore, prolonged cough (i.e., cough for more than two weeks) has a sensitivity of only 35% for TB [9]. Although individuals who experience cough may have an increased likelihood to be concerned and seek testing, those with symptoms that resolve might lack motivation to seek a consultation. This is an issue as the 2016 prevalence study conducted by the National Tuberculosis Control Program (NTP) of the Philippines showed that 14.2% of TB positive patients were asymptomatic, and only 6% of TB positive patients presented with a cough that lasted less than 2 weeks and did not belong to a high-risk group [11]. NTP definition of high-risk group included: men, elderly, those in poverty, dwellers in urban slums, smokers, previous TB patients, and diabetic individuals [11]. Therefore, while the presence of cough, most commonly identified symptom (79.7%), can be beneficial by motivating TB suspects to seek screening and testing, the lack of obvious symptoms does not necessarily signify the lack of TB infection or convalescence. The high percentage of asymptomatic TB positive patients suggests that systematic active case finding (ACF) in high prevalence areas might be required to increase case detection. With a recent rise in TB incidence (554/100,000/year) in the country, all high-risk groups in the Philippines should be considered for ACF.

International Care Ministries (ICM), a Philippine-based nongovernmental organization (NGO) that provides poverty alleviation programs to extreme low-income households, has been carrying out ACF in resource-limited settings for eight years. The intervention examined will be referred to as the “ICM TB Screening Program” (TSP) and from June 2014 to May 2015, 1290 participants were identified by this program to be TB symptom positive. In this study, we assessed how TB symptoms affected ACF outcomes in extreme low-income households.

## 2. Materials and Methods

### 2.1. ICM TB Screening Program (TSP)

**Figure 1:**
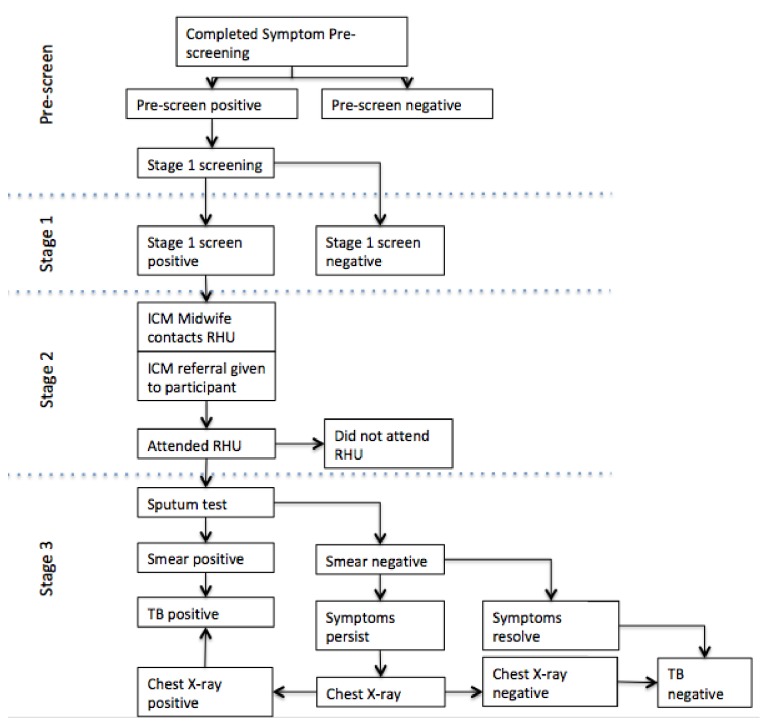
Flow chart of ICM TB Screening Program. Prescreening: over the course of 16 weeks, participants of ICM's Transform program go through poverty alleviation lessons. Participants were asked about TB physical symptoms at prescreening. Stage 1: those who were prescreened positive were asked again, the same questions, at this stage to filter out any false positive participants. Stage 2: ICM's health trainers, local staff, who are fully capable of communicating in regional dialects, refer TB suspects to the closest rural health unit (RHU) for TB testing. This is where we monitored participants' health-seeking behaviour through their decision to visit an RHU for testing or not. Stage 3: depending on the RHU, TB suspects are asked to provide a sputum sample and some would be required to have a chest X-ray taken for a more accurate diagnosis.

**Figure 2:**
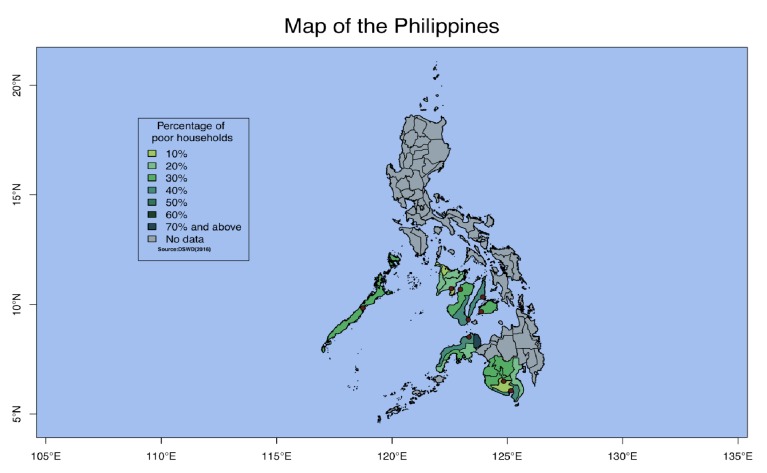
Green areas are shaded according to the percentage of poor households in provinces where ICM works in. Red dots indicate locations of ICM's offices.

The TB Screening Program (TSP) is part of ICM's 16-week poverty reduction program called Transform. This program identifies households experiencing extreme low-income using propensity scoring, and gathers approximately 30 participants in each community for a series of weekly health and livelihood lessons. These lessons are designed to equip the participants to remain healthy and to improve or secure alternative sources of income. During one of these lessons, an ICM health staff carried out ACF and facilitated follow-up in each of the communities in four stages (Figure 1). The communities that ICM works with are geographically spread out over the Visayas and Mindanao in the Philippines (Figure 2). Approximately 325 communities covering 16 different provinces are included in each cycle (16 weeks).

On week three of the Transform program, and following a health lesson on TB, all participants completed a prescreening card (Figure S1 in Supplementary Material) that gathered personal level information on TB symptoms. Adults, aged 15 and above, were categorized to be a TB suspect if they had a cough that lasted for two or more weeks. Children, 15 years and below, were suspected to have TB if found with any one of the following symptoms: (a) cough/wheezing of at least two weeks or more, (b) unexplained fever of two weeks or more, (c) significant and unintentional weight loss, (d) fatigue, lethargy, general malaise, (e) failure to respond to appropriate antibiotic therapy after two weeks, and (f) failure to regain previous state of health after two week following a presumed viral infection. Occasionally, at the ICM health staff's discretion, adults without cough but presenting with other symptoms were also referred for testing.

Individuals with TB symptoms from the prescreen progressed to Stage 1. During weeks five and six of the Transform program, patients continuing to meet the definition of suspected TB were considered Stage 1 positive. In Stage 2, a rural health unit (RHU) referral form was provided to all Stage 1 positive individuals. During weeks seven and eight, and with patient permission, ICM health staff contacted or visited each RHU to provide a list of potential TB patients identified during Stage 1. Each referred participant was asked to attend the RHU for testing. ICM staff provided TB related health knowledge and encouraged those who were hesitant or unwilling to get tested. In a few cases, transportation assistance was provided to those who were willing to visit the RHU on the days ICM staff were present in the community.

At Stage 3, and again with patient permission, ICM handed over all TB patient information to RHUs. This stage was initiated once the patient attended a RHU for sputum testing, and all patients who successfully completed the sputum test were given a RHU reply slip. Patients with a positive TB sputum result were enrolled into a directly observed treatment, short-course (DOTS) program. Patients with persistent symptoms but negative sputum results were further screened by chest X-ray (CXR). For a few participants, financial assistance was provided by ICM for the CXRs. Those determined to be TB positive by CXR were then enrolled in the DOTS program.

During weeks 9-16, RHU reply slips were returned and three kilograms of a micronutrient fortified soy-blended rice product was given to each participant who successfully attended a RHU. ICM staff followed up with those who have not visited the RHU for testing and collected results on those who were tested.

### 2.2. Data Collection

Program monitoring data was collected at each stage from every patient enrolled into the TSP. At Stage 1, potential TB patients' symptoms were recorded. Outcomes were collected by ICM staff at Stage 3 via follow-up calls with RHU staff and from reply slips completed by doctors (Figure S2 in Supplementary Material). ICM staff collected referral data on paper logbooks and updated this collected data on Google Spreadsheet (Figure S3 in Supplementary Material).

**Figure 3:**
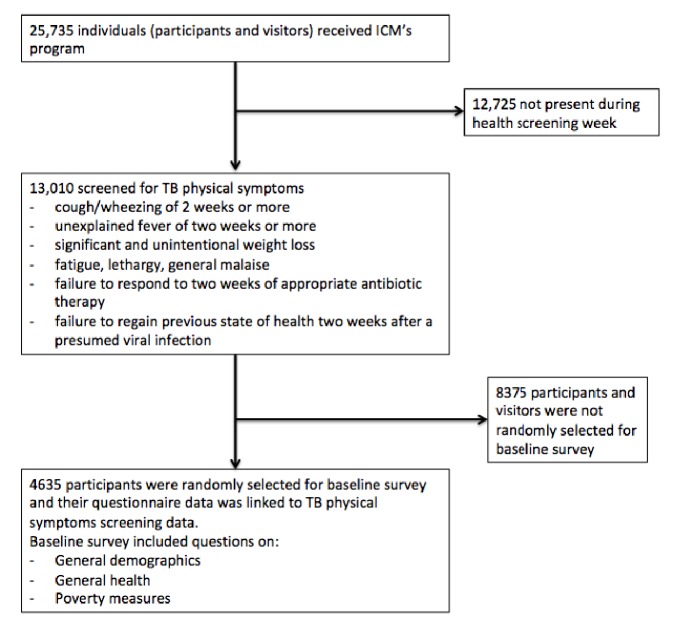
Number of participants at each step of the ACF process.

As part of ICM's program, baseline and endline questionnaires were administered at the beginning and end of the Transform program. Questions included general demographics, general health, measures of social capital, and asset-based measures of poverty. On physical symptoms, participants were asked if they had experienced: cough/wheezing of two weeks or more; unexplained fever of two weeks or more; significant and unintentional weight loss; fatigue, lethargy, general malaise; failure to respond to two weeks of appropriate antibiotic therapy; failure to regain previous state of health two weeks after a presumed viral infection. These two datasets, baseline questionnaire and physical symptoms, were linked through a unique ID tag where available. Records that could not be linked were excluded from this study. Of the 25,735 Transform program participants, who were surveyed, 13,010, including family members, were screened for TB symptoms and 4635 (36.4%) participants' records were linked (Figure 3). There was no significant difference in age, sex or RHU attendance between the group that was linked and the group that was not linked.

### 2.3. Analysis

In this study, our objective was to understand which factors were important in contributing to a successful referral which resulted in testing at a RHU. Variables such as symptoms and social capital characteristics of our study population were compared between the groups that completed testing at a RHU and of those who did not go for testing. The covariates selected for data analysis are common TB symptoms, based on Philippines NTP manual of procedures and WHO systematic screening for active TB, that were asked during our symptom screening [6, 12]. Statistical significance, defined as p<0.05, was calculated using Student's t-test and Pearson's chi-square test. To examine the effect of physical symptoms on whether a patient attended the RHU, intraclass correlations (ICC) were examined before multilevel regression modeling, to account for nesting of individuals within communities. Using the ‘MCMCglmm' package, the multilevel models were generated, and Markov chain Monte Carlo (MCMC) was used to generate p-values [13]. MCMC techniques promote convergence of such models and provide estimates of p-values and confidence intervals. All analyses were conducted using R version 3.3.3.

This study was approved by the Health Sciences Research Ethics Board of the University of Toronto (Protocol Ref #30943).

## 3. Results

### 3.1. Sociodemographics

In total, 4635 participants were screened. 1241 patients were screened from June to September 2014, 2109 patients screened from October 2014 to January 2015, and 1285 patients were screened from February to May 2015. After Stage 1 screening, 2696 were identified as TB suspects, 1290 of them were referred to a RHU, and 333 of the referred participants successfully visited a RHU for TB testing. The literacy rate of screened and referred and those who visited the RHU were similar at 53%, 55%, and 52%, respectively.

Among the individuals that visited a RHU, 189 (56%) were female participants and 147 (44%) were male participants. Additionally, 172 (51%) were registered with the national health insurance program (PhilHealth), and 53 (16%) participants tested positive for pulmonary TB (Table 1(a)). The average age of our ACF participants was 37.7 years, 38.2 years among those who were tested at a RHU, and 44.9 years among those with a confirmed TB diagnosis (see Supplementary Table S5 for breakdown by children and adults). Average household size also did not substantially differ among these three groups: 4.8, 4.6, and 5 members, respectively. Differences were noted by referral status (see Table 1(b)) for age among adults, for gender, and for PhilHealth affiliation.

### 3.2. Symptoms

Most participants reported at least one symptom (see histogram Fig S.5). Among our study population, 297 (89.2%) of the 333 study participants who were TB tested at a RHU reported having cough or wheezing for at least two weeks or more compared to a slightly lower proportion, 724 of 957 (75.7%), who failed to get tested (Table 2). Although the prevalence of other symptoms tended to be higher among those who attended the RHU (i.e., sicker participants), the differences were not statistically significant: unexplained fever of two weeks or more (6.9%; 4.6%), significant and unintentional weight loss (9.6%; 7.3%), fatigue, lethargy, general malaise (6.3%; 3.9%), failure to respond to two weeks of appropriate antibiotics therapy (2.1%; 1.7%), and failure to regain previous state of health two weeks after viral infection (0.6%; 1.7%). Of those who tested positive for TB, 45 (84.9%) were identified as having cough for two weeks or more.

On univariate analysis, only cough for two or more weeks was associated with attendance at the RHU (see Table S7). The multilevel regression results (Table 3) also showed that cough for two or more weeks was associated with successful attendance at a RHU for testing (p=0.001; OR = 1.09). The symptom of ‘failure to regain previous state of health two weeks after viral infection' was also found to be statistical relevant (p=0.032; OR=0.80). Due to the homogeneity of general demographics factors in our study population, and despite inclusion of these factors and additional social capital factors in the multilevel regression modeling, we did not find them statistically significant.

## 4. Discussion

One of the biggest challenges faced by our study participants is access to health services. ICM has continuously carried out active case finding over the last eight years in approximately 900 communities each year, and accessibility to health facilities has consistently been a challenge. For our study participants who live in a low socioeconomic position, this challenge may be made up of different factors including distance from the RHU, stigma associated with TB, lack of financial resources, inability to take sick leave, and restricted hours of health service operation. This study explored the physical symptoms that extreme low-income Filipinos identified to influence health service-seeking behaviour.

### 4.1. Limitations in the System

From this experience, we find that passive case finding systems alone, where a TB symptomatic individual presents himself/herself to a healthcare facility, would not adequately capture suspected TB cases in high prevalence and resource-limited settings. Even for those who make it to the RHU for testing, their diagnosis might not be accurate. For our study participants, sputum smear microscopy is commonly used to provide preliminary information for a TB diagnosis. While the cost for this diagnostic test is low, it also has a low sensitivity (0.61). Additionally, those participants who were diagnosed using sputum smear microscopy were asked to return another day to receive their results. Thus, receiving a positive TB diagnosis often involves taking at least two days off from work and finding money for transportation to make two trips to the RHU. Past studies have shown that negative stigmatization of TB exists in the Philippines [12]. Since TB symptom screening was conducted before education on TB, stigma may have prevented more people from disclosing their TB symptoms.

### 4.2. Prolonged Cough as a Symptom of TB

Based on our findings, it could be inferred that the study participants associated coughing with TB. Prolonged cough was the only physical symptom found to be significantly different between those who were referred and tested, and those who were referred and not tested. A similar finding was reported by a study conducted in Palawan, Philippines, with a larger study population which included 12907 rural poor, 1625 urban poor, and 2145 indigenous people [14]. Among their TB positive participants, 53.9% reported having cough (of any duration) and 25.8% reported having cough for more than two weeks. Comparatively in our study, 85% (45 out of 53) of subjects who tested positive for TB infection presented with prolonged cough. In our study the odds ratio of a referred participant visiting a health centre for TB testing due to prolonged cough symptom was smaller than expected.

Since this ACF was designed based on the most up-to-date symptom screening guidelines of Philippines' National Tuberculosis Control Program in 2013, the primary algorithm referred those who coughed for more than two weeks to a RHU for TB testing. Though ICM staff asked about other symptoms of TB during the screening process, those without prolonged cough were not referred to the RHU for testing.

Cough for two weeks or more was also the most common symptom (79.7%) identified by those presenting any TB symptom in the 2016 survey [2]. Only 2815 (4.82%) of all the NTP 2016 survey participants presented any TB symptom, and of these participants, 2250 (80%) of them had cough for two weeks or more. Of those who were TB confirmed, 298 (64%) did not present prolonged cough, but were CXR positive.

### 4.3. Expected vs. Observed ACF Results

According to the WHO guidance on planning and implementation of systematic screening for active TB (online tool), not taking our population's relatively higher risk into account, utilizing an algorithm of cough for two or more weeks as the primary symptom and microscopy as the diagnostic tool, we can expect to only identify 20% of all TB cases. This would rise to approximately 38% if Xpert®MTB/RIF (Xpert) is used as the diagnostic tool instead [6].

However, the global detection rate for TB has not significantly improved even with the recent dissemination of Xpert [15]. Inequity in access and dissemination of this new technology is partly responsible, as well as the prevalence of asymptomatic and latent TB cases which has limited drastic improvements. Limitations in access are more pronounced among those who face geographical and socioeconomic barriers [16]. Despite efforts made by the Philippine Department of Health (DOH) and various international funding agencies to make Xpert readily accessible to Filipinos, the participants of this study were mostly unable to benefit from this progress.

We observed a higher prevalence of TB confirmed in the communities included in this study compared to the national rate (approximately 1%). We speculate that the high prevalence is due to our study participants experiencing extreme low-income, which is considered to be a high-risk group for TB in the Philippines. The prevalence in our study population, 53 confirmed cases out of 333 TB tested (15.8%), is substantially higher than that of the study conducted in Palawan (2.2% for rural poor and 2.1% for the urban poor) [14].

Given our study population's difficulty accessing diagnosis with an Xpert, the majority of our participants would have been diagnosed using sputum smear microscopy. This implies that we might have only detected 21% of true positive TB cases or fewer among those who were referred to a RHU for testing and were tested using sputum smear microscopy, based on the WHO's estimation on systematic screening for active TB. Other studies from high prevalence countries have shown that populations experiencing low-income are about 4.5–5.5 times or more vulnerable to TB infection [17–19]. However, with the low testing proportion in our study population, case detection numbers are low [20]. This means that if ACF was performed using Xpert, then there is a possibility that more TB cases would have been detected. Lastly, in the Philippines, HIV prevalence is low at 0.1 for adults, age 15 to 49 [21]. Perhaps this could be another reason why the testing and case detection rate is low. About 54% of Human Immunodeficiency Virus (HIV) positive Filipinos are co-managing HIV and TB [21]. We did not collect HIV/AIDS status of our participants.

## 5. Conclusion

Based on our study, prolonged cough was recognized as a TB symptom by those in geographically isolated, poor communities, possibly motivating these participants to get tested. Though the odds ratio was only 1.09 (95% CI 1.08, 1.25), we believe that it is still a meaningful finding from our study participants as this could be due to the low proportion of TB patients with noticeable symptoms. ACF strategies in high-risk groups need to be revised so asymptomatic patients are not inadvertently overlooked due to lack of cough. Currently, greater effort is being made to find asymptomatic TB cases by treating latent TB patients globally [22]. Since cough is already recognized as a TB symptom, health promotion activities should aim to educate the public on the high percentage of asymptomatic cases in order to encourage testing even in the absence of prolonged cough. Relying solely on cough as a symptom is precarious since this symptom is highly dependent on severity, and is subjective to each individual's tolerance level. Additionally, detection through symptom screening, prolonged cough, has low sensitivity of 0.247 [6]. Though the results from this paper cannot be generalized for the whole of Philippines, they provide a good understanding of problems that need to be addressed to detect missing TB cases among individuals experiencing low-income, in order to achieve national targets. The study was not able to understand the transmission among close contact groups with TB confirmed participants. ACF using mobile diagnostics tools, widespread screening of high-risk populations, and sputum sample transportation service are possible options for increasing efficiency of ACF in hard to reach areas.

## Figures and Tables

**Table:** (a) General demographics at each step of the TB Screening Program.

	Stage 1: Suspected Cases (N= 2696)		Stage 2: Needed Referral (N=1290)		Stage 3: Attended RHU (N=333)		TB Positive (N=53)	
	n	(%)	n	(%)	n	(%)	n	(%)
% of prior stage	2696/4635	58.2	1290/2696	47.8	333/1290	26	53/333	15.9
Female	1476	0.6	689	0.5	187	0.6	28	0.5
Male	1220	0.5	601	0.5	146	0.4	25	0.5
Registered in PhilHealth^1^	1420	0.5	694	0.5	172	0.5	30	0.6

	Mean	(SD)	Mean	(SD)	Mean	(SD)	Mean	(SD)

Average Age	37.7	21.4	36.5	22.4	38.2	23.2	44.9	18.5
Average Household Size	4.8	2.4	4.8	2.4	4.6	2.5	5	2.8
Average Household Income (PHP per person per day)	22.9	25.8	20.4	20.3	20.7	22.1	18.6	10.2

^1^PhilHealth is the Philippine government-owned nation health insurance program.

**Table:** (b) Demographic characteristics of referred and not referred participants

Characteristic	Referred RHU (n = 1290)		Not referred (n = 1406)		P-value	Significance*∗*
	mean	SD	mean	SD	For difference in means	
Age	36.5	22.9	27.3	23.1	<0.001	*∗∗∗*
Child	5.4	4.0	5.2	3.8	0.6338	
Adult	46.6	16.5	43.5	17.2	<0.001	*∗∗∗*

HH.size	4.8	2.4	4.9	2.2	0.1859	
HH.income	2649.3	2570.6	3353.6	3571.6	<0.001	*∗∗∗*

	Number	Denominator	Number	Denominator	For difference in proportions	

Male	601	2116	1515	2116	<0.001	*∗∗∗*
Female	689	2519	1830	2519	<0.001	*∗∗∗*
Phil. health	694	4635	1865	4635	<0.001	*∗∗∗*

*∗*P value of <0.001 was considered to be statistically highly significant.

**Table 2:** TB symptoms and signs of study participants divided by those who attended RHU for TB testing and those who did not attend RHU and did not get tested for TB.

	Attended RHU (N=333)		Did Not Attend RHU (N=957)		
TB Symptoms & Signs	n	(%)	n	(%)	p-value^1^
Cough/Wheezing of 2 weeks or more	297	89.2	724	75.7	<0.001
Unexplained fever of 2 weeks or more	23	6.9	44	4.6	0.14
Significant and unintentional weight loss	32	9.6	70	7.3	0.22
Fatigue, lethargy, general malaise	21	6.3	37	3.9	0.09
Failure to respond to 2 weeks of appropriate antibiotic therapy	7	2.1	16	1.7	0.79
Failure to regain previous state of health 2 weeks after viral infection	2	0.6	16	1.7	0.24

^1^P-values calculated using Chi-sq test.

**Table 3:** Factors associated with successful attendance at a Rural Health Unit (Markov chain Monte Carlo (MCMC) generalized linear mixed-model).

Outcome: Attended RHU	Posterior mean (95% CI)	Odds Ratio (95% CI)	p-value
*Fixed*			
(Intercept)	0.16 (0.08, 0.22)	1.17 (1.08, 1.25)	<0.001
Cough/Wheezing of 2 weeks or more	0.08 (0.01, 0.14)	1.09 (1.01, 1.15)	0.01
Unexplained fever of 2 weeks or more	0.01 (-0.10, 0.11)	1.01 (0.91, 1.12)	0.78
Significant and unintentional weight loss	0.03 (-0.06, 0.12)	1.03 (0.94, 1.12)	0.55
Fatigue, lethargy, general malaise	0.05 (-0.06, 0.18)	1.06 (0.94, 1.20)	0.40
Failure to respond to 2 weeks of appropriate antibiotic therapy	0.14 (-0.05, 0.32)	1.14 (0.95, 1.37)	0.15
Failure to regain previous state of health 2 weeks after viral infection	-0.22 (-0.43, -0.03)	0.80 (0.65, 0.97)	0.03
*Random*			

*σ* (community)	0.05 (0.01,0.11)		
*σ* (base)	0.03 (0.02, 0.04)		
Deviance (DIC)			1278.30

## Data Availability

The data used to support the findings of this study are available from the corresponding author upon request if permission is granted from the Health Sciences Research Ethics Board of the University of Toronto.

## Additional Points

*Study Limitations. *The authors acknowledge that more statistically significant findings could have been discovered if the study sample size was larger. Through this study we discovered that participants experiencing extreme poverty in the Philippines could be associating cough for 2 or more weeks as a symptom of TB. However, this study was not designed to answer that question and it will be followed up with a more in-depth qualitative study to understand study population's knowledge, attitudes, and practices for health seeking regarding TB.

## Supplementary Materials

Supplementary Materials Four figures and three tables have been provided under the Supplementary Materials to provide visual aid for clearer understanding of the study. Figure S1, S2, and S3 were added to show the forms used for data collection. Table S4 was added as a response to a comment by a reviewer. Table S4 shows the literacy rate of our participants. Table S5 was also added based on a reviewer's comment to show the percentage of adults and children at each stage. Figure S6 shows that having one symptom was the most common among our study participants. Table S7 was added to show univariate analysis of all TB symptoms used during TB screening of the study.
