# Supplementary material for: The Presence of Cough and Tuberculosis: Active Case Finding Outcomes in the Philippines
Source: Tuberc Res Treat. 2019 Aug 21;2019:4578329. doi: 10.1155/2019/4578329 (PMC6719270; doi:10.1155/2019/4578329)
Supplement: Supplementary Materials — Four figures and three tables have been provided under the Supplementary Materials to provide visual aid for clearer understanding of the study. Figure S1, S2, and S3 were added to show the forms used for data collection. Table S4 was added as a response to a comment by a reviewer. Table S4 shows the literacy rate of our participants. Table S5 was also added based on a reviewer's comment to show the percentage of adults and children at each stage. Figure S6 shows that having one symptom was the most common among our study participants. Table S7 was added to show univariate analysis of all TB symptoms used during TB screening of the study. [file 4578329.f1.pdf]

Supplementary Materials

Figure S1. ICM Health Check Card

ICM

Health Check Card (June 2014)

Participant name: ______________________ Trainer: ____________________

Community/ID: ______________________ Pastor: ______________ Batch: ________

Adult: 15 years old and above	Name of adult with cough	Age	Name of adult with cough	Age
Do you or any adult in your household have cough for more than 2 weeks?				

Children: 0 -14 years old	Name of child with symptoms	Age	Name of child with symptoms	Age
Is there a child or children in your household that has any of the following symptoms?				
	Check the symptoms that the child has		Check the symptoms that the child has	
1. Cough for more than 2 weeks	☐		☐	
2. Fever for more than 2 weeks	☐		☐	
3. Significant/unintentional weight loss	☐		☐	
4. Fatigue, lethargy, general malaise	☐		☐	
5. Failure to respond to appropriate antibiotic therapy after two weeks	☐		☐	
6. Failure to regain previous state of health after two week following a presumed viral infection	☐		☐	

Figure S2. ICM RHU referral card

ICM

ICM – RHU REFERRAL CARD

To: ______________________ Date of Referral: _________

(Name of Facility)

Reason for Referral (symptom):______________________

____

Name of Patient: ____________________________________

Present Address:______________________________________

Age: _______ Sex: ☐ Male ☐ Female

Patient contact no: ___________________________________

Referred By: ____

-✂---

ICM Return Slip

Name of RHU/MHU staff: ____________________________

Designation: ____________Date Received: _____________

Patient: ____

Diagnosis: _____________________ Sputum AFB? Yes / No

Treatment:____

Figure S3. TB follow-up sheet used by ICM staff to collect data on TB symptom positive participants

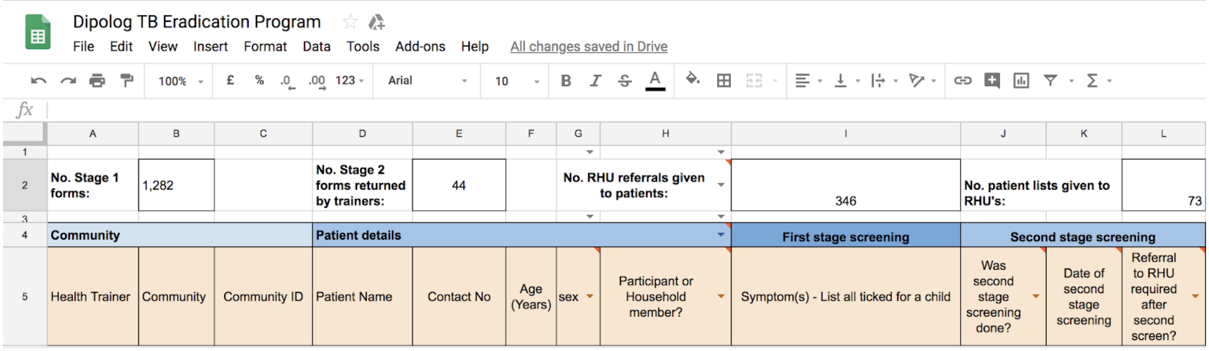
Dipolog TB Eradication Program
File Edit View Insert Format Data Tools Add-ons Help All changes saved in Drive
No. Stage 1 forms:
1,282
No. Stage 2 forms returned by trainers:
44
No. RHU referrals given to patients:
346
No. patient lists given to RHU's:
73
Community
Patient details
First stage screening
Second stage screening
Health Trainer
Community
Community ID
Patient Name
Contact No
Age (Years)
sex
Participant or Household member?
Symptom(s) - List all ticked for a child
Was second stage screening done?
Date of second stage screening
Referral to RHU required after second screen?

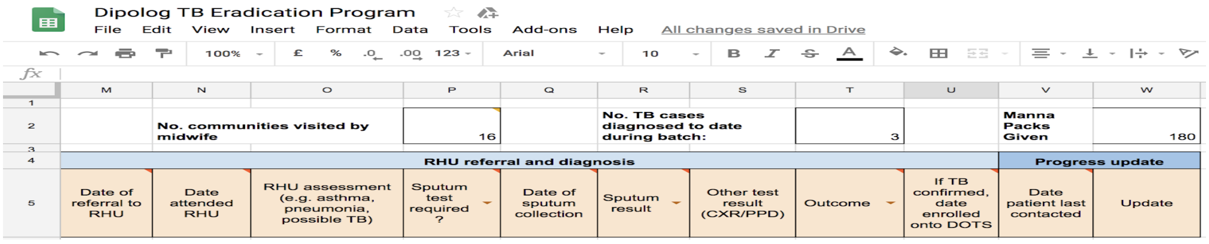
Dipolog TB Eradication Program
File Edit View Insert Format Data Tools Add-ons Help All changes saved in Drive
No. communities visited by midwife
16
No. TB cases diagnosed to date during batch:
3
Manna Packs Given
180
RHU referral and diagnosis
Progress update
Date of referral to RHU
Date attended RHU
RHU assessment (e.g. asthma, pneumonia, possible TB)
Sputum test required ?
Date of sputum collection
Sputum result
Other test result (CXR/PPD)
Outcome
If TB confirmed, date enrolled onto DOTS
Date patient last contacted
Update

Table S4. Literacy rate among program participants

Literacy	Screened	%	Referred	%	Attended	%
Illiterate	1708	36.85%	420	32.56%	109	32.73%
Literate	2441	52.66%	707	54.81%	172	51.65%
N/A*	486	10.49%	163	12.64%	52	15.62%

*participant data was absent

Table S5. Children versus adults at each stage

Category	Screened	%	Referred	%	Attended	%
**Children**	1708	36.85%	315	24.42%	81	24.32%
**Adult**	2863	61.77%	968	75.04%	248	74.47%
**N/As***	64	1.38%	7	0.54%	4	1.20%

*some participants' age were missing from the data set.

Figure S6. Histogram of participants identified with different

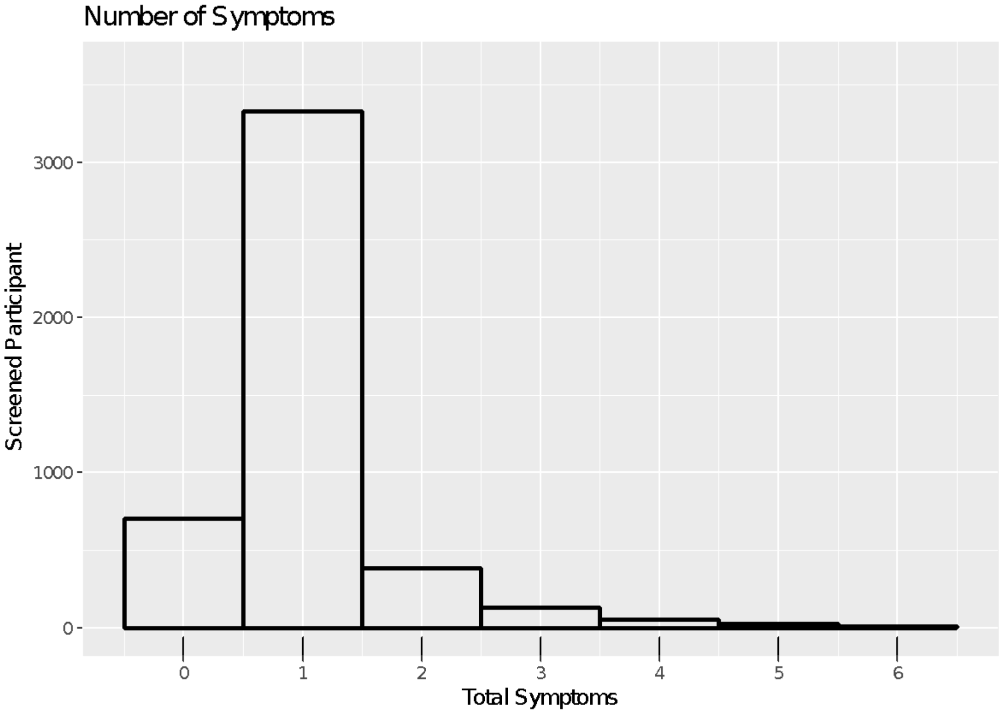

Table S7. Univariate analysis of all symptom variables

Coefficients	Estimate	Std. Error	Pr(>|t|)	
**(Intercept)**	0.13	0.03	<0.001	***
**cough.2wks**	0.16	0.03	<0.001	***

**AIC: 1507**				

Coefficients	Estimate	Std. Error	Pr(>|t|)	
**(Intercept)**	0.25	0.01	<0.001	***
**fever.2wks**	0.09	0.05	0.102	

AIC: 1532				

Coefficients:	Estimate	Std. Error	Pr(>|t|)	
(Intercept)	0.25	0.01	<0.001	***
weight.loss	0.06	0.05	0.182	

AIC: 1532.9				

Coefficients:	Estimate	Std. Error	Pr(>|t|)	
(Intercept)	0.25	0.01	<0.001	***
gen.malaise	0.11	0.06	0.0643	.

AIC: 1531.3				

Coefficients:	Estimate	Std. Error	Pr(>|t|)	
(Intercept)	0.26	0.01	<0.001	***
antibiotics.2wks	0.05	0.92	0.61	

AIC: 1534.4				

Coefficients:	Estimate	Std. Error	Pr(>|t|)	
(Intercept)	0.26	0.01	<0.001	***
virus.2wks	-0.15	0.10	0.151	

AIC: 1532.6				
